# Metallic protection of soil carbon: divergent drainage effects in *Sphagnum* vs. non-*Sphagnum* wetlands

**DOI:** 10.1093/nsr/nwae178

**Published:** 2024-05-20

**Authors:** Chengzhu Liu, Yunpeng Zhao, Lixiao Ma, Guoqing Zhai, Xingqi Li, Chris Freeman, Xiaojuan Feng

**Affiliations:** State Key Laboratory of Vegetation and Environmental Change, Institute of Botany, Chinese Academy of Sciences, Beijing 100093, China; China National Botanical Garden, Beijing 100093, China; University of Chinese Academy of Sciences, Beijing 100049, China; State Key Laboratory of Vegetation and Environmental Change, Institute of Botany, Chinese Academy of Sciences, Beijing 100093, China; China National Botanical Garden, Beijing 100093, China; University of Chinese Academy of Sciences, Beijing 100049, China; State Key Laboratory of Vegetation and Environmental Change, Institute of Botany, Chinese Academy of Sciences, Beijing 100093, China; China National Botanical Garden, Beijing 100093, China; University of Chinese Academy of Sciences, Beijing 100049, China; State Key Laboratory of Vegetation and Environmental Change, Institute of Botany, Chinese Academy of Sciences, Beijing 100093, China; China National Botanical Garden, Beijing 100093, China; University of Chinese Academy of Sciences, Beijing 100049, China; State Key Laboratory of Vegetation and Environmental Change, Institute of Botany, Chinese Academy of Sciences, Beijing 100093, China; China National Botanical Garden, Beijing 100093, China; University of Chinese Academy of Sciences, Beijing 100049, China; School of Natural Sciences, Bangor University, Bangor LL57 2UW, UK; State Key Laboratory of Vegetation and Environmental Change, Institute of Botany, Chinese Academy of Sciences, Beijing 100093, China; China National Botanical Garden, Beijing 100093, China; University of Chinese Academy of Sciences, Beijing 100049, China

**Keywords:** wetland drainage, vegetational change, metal-organic associations, carbon sequestration

## Abstract

The established paradigm assumes that drainage may decrease the vast soil organic carbon (SOC) reservoir in global wetlands. Yet drainage can also promote SOC stabilization by fostering the accrual of metal-bound organic carbon (bound OC) upon oxygen exposure. Here, this emergent mechanism is tested for the first time at a regional scale, using literature data and a nationwide, pairwise survey of drained wetlands across China. We show that long-term (15–55 years) drainage largely increased metallic protection of SOC (bound OC%) in non-*Sphagnum* wetlands, but consistently decreased bound OC% in *Sphagnum* wetlands following replacement of the ‘rust engineer’ *Sphagnum* by herbaceous plants. Improved SOC stock estimates based on 66 soil profiles reveal that bound OC increases can compensate for the loss of unbound SOC components in non-*Sphagnum* wetlands with substantial accrual of reactive metals. Metallic stabilization of wetland SOC is hence a widespread but overlooked mechanism that is heavily influenced by vegetational shifts. Incorporating this novel mechanism into models will improve prediction of wetland SOC dynamics under shifting hydrological regimes.

## Teaser

Drainage enhances metallic protection of soil carbon in non-*Sphagnum* wetlands in contrast to *Sphagnum* wetlands due to varied vegetational shifts.

## INTRODUCTION

Wetlands represent vast terrestrial carbon reservoirs (storing ca. 600–1000 Gt C) [1–3] that play a pivotal role in the feedback between carbon cycle and climate change [2,4]. Approximately 21% of the global wetland area (ca. 3.4 million km^2^) has been lost to human-induced drainage for agriculture and pasture between 1700 and 2020 [5,6], severely threatening wetland carbon sink capacity [7,8]. However, the response of wetland soil organic carbon (SOC) stocks to drainage remains uncertain, with reports of negative, neutral and even positive changes [9–12]. This uncertainty reflects the complexity, as well as our limited understanding, of the processes and mechanisms regulating wetland soil carbon sequestration under shifting hydrological regimes [10]. Deciphering the mechanisms that mediate the contrasting responses of wetland SOC to drainage is pivotal to accurately understanding and predicting wetland carbon dynamics under a changing climate.

The current paradigm assumes that wetland SOC primarily consists of poorly decomposed plant detritus in the form of particulate organic carbon (POC) [13,14] that is subject to rapid decomposition upon drainage and oxygen exposure [15]. However, recent studies suggest that organic carbon (OC) bound to reactive (i.e. poorly crystalline or short-range-ordered, SRO) iron (Fe) and aluminum (Al) (hydr)oxides (abbreviated as ‘bound OC’ hereafter) constitutes a sizable fraction (up to 40%) of SOC in the mineral layers of wetlands [16–20]. This OC fraction has a longer turnover time than POC [21,22] and may govern the long-term persistence of wetland SOC. Furthermore, contrary to the postulated rapid loss of POC, bound OC may accumulate after drainage owing to an increased protection of organic matter by newly formed reactive Fe (hydr)oxides via ferrous Fe (Fe(II)) oxidation (i.e. the ‘iron gate’ mechanism) [23]. The potential accrual of bound OC may thus compensate for, or even override, POC loss at long timescales and underpin SOC stability in wetlands. However, this emergent Fe-mediated SOC accumulation mechanism has not been tested at a regional scale [23–27]. The prevalence and magnitude of increases in bound OC await confirmation in different wetlands and along soil depths to determine their influence on wetland SOC variations following drainage.

Compared with vascular plant-dominated (non-*Sphagnum*) wetlands, *Sphagnum* wetlands exhibit distinct reactivity of metal oxides and edaphic properties [19,28–30]. As a well-known ‘peat builder’ [6,31,32], *Sphagnum* dominates more than half of northern wetlands [33,34], which hold ∼15% of the soil carbon pool globally [35]. It was recently shown that *Sphagnum* greatly promotes the formation of SRO Fe and Al (hydr)oxides owing to its acidic, phenolic metabolites [29,36] and acts as an efficient ‘rust engineer’ to increase soil's sequestration capacity of bound OC [19]. However, *Sphagnum* is sensitive to water stress and subject to replacement by vascular plants following drainage [37–39]. The stability and dynamics of the remarkable pool of bound OC in *Sphagnum* wetlands thus warrant particular attention. With diminishing *Sphagnum* and its acidic, phenolic metabolites after drainage, we hypothesize that Fe-OC interaction may weaken, leading to decreasing bound OC and SOC in *Sphagnum* wetlands, in contrast to non-*Sphagnum* wetlands. Verifying the potential contrasting responses of bound OC to drainage in different wetlands would constrain the relevance of metallic protection in regulating wetland SOC changes and aid identification of priority areas and strategies for wetland carbon protection and restoration.

**Figure 1.**
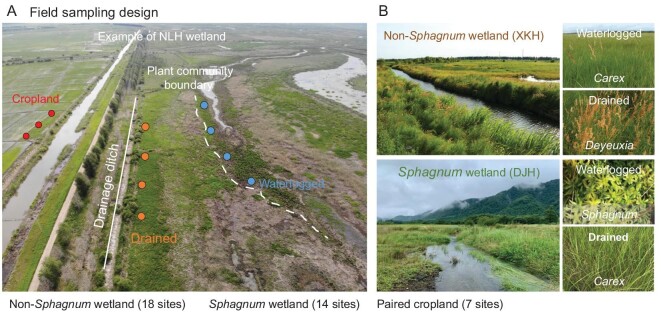
Overview of sampling design and study sites. (A) Field sampling design. (B) Pictures of drainage ditches and changes in plant community after decades of artificial drainage at two sites.

To test our hypothesis, we conducted a large-scale, pairwise survey of typical wetlands across China, spanning gradients of climate, geology and edaphic characteristics (Fig. 1 and Table S1). The survey involved replicated pairs (*n* = 4) of waterlogged vs. drained (by ditching for 15–55 years) soils in 32 wetlands, including 18 non-*Sphagnum* and 14 *Sphagnum* wetlands. Most of our study sites (24 of 32) were located in Natural Reserves, where the drained area had not been reclaimed after ditch construction. We first examined drainage-induced changes of SOC and bound OC in the surface soils (0–20 cm) of all surveyed wetlands. To supplement our data set, we also compiled published SOC changes in drained wetlands elsewhere. Given the large number of related studies based on reclaimed soils, we separated different land-use types (i.e. ‘drainage only’ and ‘reclamation after drainage’) to examine their SOC responses to wetland drainage. Second, to investigate drainage effects along depths, we analyzed SOC and bound OC variations along soil profiles (mostly 0–50 cm) in 11 pairs (*n* = 3) of our surveyed wetlands, involving 66 soil cores. In total, our analysis entailed 438 soil samples. We further examined plant biomass, soil properties (pH and soluble phenols) and metal species (Fe(II), reactive Fe and Al (hydr)oxides) that potentially regulated bound OC in soils, and employed pathway analysis to disentangle the cascading effects of drainage on bound OC in different wetlands. With these approaches, we aimed to compare and contrast drainage-induced metallic protection of SOC in *Sphagnum* vs. non-*Sphagnum* wetlands and to reveal the underlying mechanisms related to SOC preservation under shifting wetland hydrology.

## RESULTS AND DISCUSSION

### Divergent responses of bound OC to wetland drainage

To investigate the response of bound OC to long-term drainage, we surveyed 32 pairs of waterlogged vs. drained soils (0–20 cm) in typical wetlands across China with ditch constructions (Fig. 1, Table S1 and Data S1). The distance between the drained and waterlogged areas of each wetland varied between 100 and 800 m. The water-table declined to 20–100 cm below soil surface (denoted as −20 to –100 cm) after 15–55 years of drainage in the drained area, but remained above soil surface in the waterlogged area (Table S1). Drainage shifted the dominant species from plants such as *Carex* and *Polygonum* in the waterlogged area, to plants including *Carex, Deyeuxia* and *Kobresia* in the 18 non-*Sphagnum* wetlands, and from *Sphagnum* to plants such as *Carex* and *Deyeuxia*, in the 14 *Sphagnum* wetlands (Table S1).

**Figure 2.**
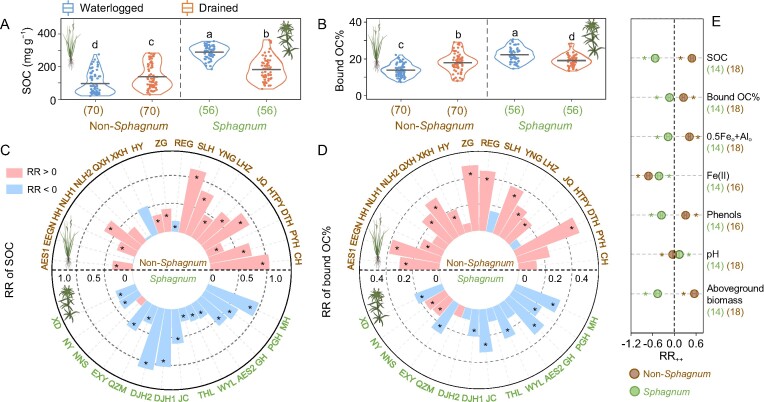
Contrasting responses of metal-organic associations and edaphic properties to drainage among non-*Sphagnum* and *Sphagnum* wetlands. (A and B) SOC contents and bound OC%. (C and D) Response ratio (RR) of SOC and bound OC% in the drained relative to waterlogged soils for non-*Sphagnum* and *Sphagnum* wetlands, with the bar size indicating the absolute value of RR. (E) Site-weighted response ratio (RR_++_) for non-*Sphagnum* and *Sphagnum* wetlands to drainage. SOC, soil organic carbon; bound OC%, proportion of organic carbon bound to reactive metal oxides in SOC; 0.5Fe_o_ + Al_o_, weight-normalized contents of oxalate-extractable iron (Fe_o_) and aluminum (Al_o_); Fe(II), ferrous iron extracted by hydrochloric acid. Lowercase letters in (A) and (B) indicate different levels (*P* < 0.05; by linear mixed effects models, with site served as random factor). Shapes of the violin represent distribution of the corresponding data. The dots and solid lines in the violin plot denote the raw data and mean of each data set, respectively. Numbers in parentheses indicate the number of samples. Asterisks in (C) and (D) denote significant difference between drained and waterlogged wetlands (*P* < 0.05; one-way ANOVA). Circles in (E) represent values of RR_++_ and bars represent the 95% CIs. If 95% CI of RR_++_ does not overlap with zero, the response is considered to be significant and marked with an asterisk.

The waterlogged *Sphagnum* wetlands had a higher surface SOC content (288.8 ± 5.4 mg g^−1^; mean ± SE; *n* = 56), bound OC content (64.1 ± 1.9 mg g^−1^; *n* = 56) and higher proportion of bound OC in SOC (denoted as ‘bound OC%’; 22.2 ± 0.5%; *n* = 56; determined by the citrate-bicarbonate-dithionite (CBD) method) [40] than the waterlogged non-*Sphagnum* wetlands (96.5 ± 8.0 mg g^−1^, 14.5 ± 1.4 mg g^−1^ and 13.9 ± 0.4%, respectively; *n* = 70; *P* < 0.05; Fig. 2A and B; Data S1). The bound OC% values indicated that SRO Fe and Al (hydr)oxides played a more important role in SOC protection in the *Sphagnum* than non-*Sphagnum* wetlands. Surface SOC content increased after drainage relative to the waterlogged areas in most (13 out of 18) of the non-*Sphagnum* wetlands, but decreased in most (13 out of 14) of the *Sphagnum* wetlands (Fig. 2A and C, and Fig. S1A). Similarly, bound OC% increased in 12 out of 18 non-*Sphagnum* wetlands with no changes in the rest (Fig. 2B and D, and Fig. S1B); but decreased in 8 out of 14 *Sphagnum* wetlands, increased at two sites (NNS and NY), and did not change at the remaining four sites (Fig. 2B and Fig. S1B). These results suggested that drainage-induced soil carbon stabilization by reactive metals [23] was prevalent in our investigated non-*Sphagnum* wetlands, whereas this mechanism seemed to be weak or absent in the *Sphagnum* wetlands after drainage.

To facilitate comparisons between the non-*Sphagnum* and *Sphagnum* wetlands, we further examined data on variables potentially related to bound OC variations, including weight-normalized content of oxalate-extractable Fe and Al (i.e. 0.5Fe_o_ + Al_o_; representing poorly crystalline or SRO phases) [41,42], acid-extractable ferrous iron (Fe(II); the precursor and reduction product of SRO Fe (hydr)oxides) [29,43], soluble phenols (with a high affinity to SRO Fe and Al (hydr)oxides) [44], soil pH [45], and plant aboveground biomass (indictive of plant productivity or input) [44]. We calculated the response ratio (RR) of bound OC% and the related variables as the natural logarithm-transformed ratio of a specific variable in the drained area relative to the waterlogged area of each wetland (Fig. 2C and D). A positive value of RR indicated an increase of the examined variable in the drained area than waterlogged area, and vice versa. Given different variances of the calculated RR at different sites, the weight of each site was estimated based on the reciprocal of the variance for individual RR [46]. We evaluated site-weighted response (RR_++_) by weighting the RR of an individual site with its inverse variance (see details in Methods) for the non-*Sphagnum* and *Sphagnum* wetlands, respectively.

According to the RR_++_, drainage decreased the surface soil Fe(II) content of both types of wetland (*P* < 0.05; Fig. 2E), reflecting Fe(II) oxidation under oxygen exposure. However, the rest of the examined variables showed contrasting responses to drainage in the non-*Sphagnum* vs. *Sphagnum* wetlands (Fig. 2E). In line with the ‘iron gate’ mechanism [23], drainage decreased soil pH (due to Fe(II) oxidation) [47] and increased SRO Fe and Al (i.e. 0.5Fe_o_ + Al_o_), bound OC% and SOC content of the non-*Sphagnum* wetlands. Drainage also increased plant aboveground biomass and soluble phenols in the soil. By contrast, drainage increased soil pH and decreased plant aboveground biomass, soluble phenols and 0.5Fe_o_ + Al_o_ in the *Sphagnum* wetlands, likely due to the replacement of the acid- and phenol-producing ‘rust engineer’ *Sphagnum* [29,36] by herbaceous plants (such as *Carex* and *Deyeuxia*). As a result, both bound OC% and SOC contents decreased after drainage in the *Sphagnum* wetlands. These results supported our hypothesis and suggested divergent response of bound OC to drainage in the non-*Sphagnum* vs. *Sphagnum* wetlands, which may closely relate to the contrasting changes of soil properties and plant communities.

### Pathways regulating bound OC responses

To ascertain factors regulating bound OC% variation following drainage, we first examined correlations between bound OC% and environmental variables. Bound OC% was strongly correlated with 0.5Fe_o_ + Al_o_ (r = 0.79; *P* < 0.001), followed by pH (r = –0.56; *P* < 0.001), soluble phenols (r = 0.55; *P* < 0.001) and Fe(II) content (r = 0.31; *P* < 0.001) in all wetlands (Fig. S2A–F). Plant aboveground biomass was also positively correlated with bound OC% in the non-*Sphagnum* wetlands (r = 0.46; *P* < 0.01; Fig. S2G). However, the relationship was absent after the effect of 0.5Fe_o_ + Al_o_ was controlled for by partial correlation analysis (Table S2). Random forest analysis confirmed that 0.5Fe_o_ + Al_o_ was the most important factor influencing bound OC% in both non-*Sphagnum* and *Sphagnum* wetlands, followed by soluble phenols, Fe(II) and soil pH (Fig. S2E and F).

**Figure 3.**
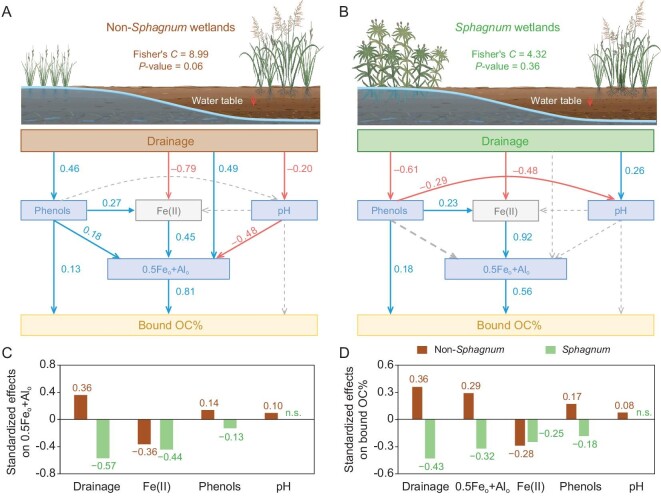
Pathways driving bound OC% changes after wetland drainage and the effect size of environmental variables. (A and B) Structural equation models (SEMs) showing the direct and indirect effects of wetland drainage on bound OC% in (A) non-*Sphagnum* (*n* = 128) and (B) *Sphagnum* (*n* = 112) wetlands. (C) Effect size of overall drainage, Fe(II), soluble phenols and pH on 0.5Fe_o_ + Al_o_. (D) Effect size of overall drainage, 0.5Fe_o_ + Al_o_, Fe(II), soluble phenols and pH on bound OC%. Abbreviations are defined in Fig. 2. Solid or dashed lines indicate significant (*P* < 0.05) or non-significant relationships (*P* > 0.05). Numbers near the pathway arrow indicate the standard path coefficients.

To further disentangle the direct and indirect pathways driving bound OC variations, structural equation modeling (SEM) was constructed with an a priori model (Fig. S3) based on knowledge [29,36,41,44,48] and the aforementioned correlations (Fig. S2A–F). Soil pH, 0.5Fe_o_ + Al_o_ and soluble phenols were direct regulators of bound OC% (Fig. 3A and B), as metal-OC interactions are known to strengthen with decreasing pH [45,49] and increasing abundance of phenolic compounds [50,51] as well as SRO Fe and Al (hydr)oxides [19,20]. Drainage directly affected soil pH, soluble phenols and Fe(II), all of which may influence 0.5Fe_o_ + Al_o_ [29,43,44]. A direct influence of drainage on 0.5Fe_o_ + Al_o_ was also included to account for drainage effects on other non-investigated soil properties/processes that potentially affected SRO Fe and Al, such as Fe-reducing microbes and soil aggregation. [16,42,52]. Soluble phenols and pH may influence Fe(II) by affecting the dissolution, complexation and speciation of Fe in the soil [29].

The validated SEMs yielded a good model fit for both non-*Sphagnum* and *Sphagnum* wetlands, indicated by the Fisher's *C*-test (0.05 < *P* < 1.00). Based on the SEM, 0.5Fe_o_ + Al_o_ exerted a dominant, direct effect on bound OC% in both types of wetlands (Fig. 3A and B; standardized path coefficient, *b* = 0.81 and 0.56, respectively), followed by soluble phenols. Soil pH had no significant direct influence. In the non-*Sphagnum* wetlands, drainage had an overall positive effect on 0.5Fe_o_ + Al_o_ and hence bound OC%, also via increasing soluble phenols and decreasing soil pH, despite decreasing Fe(II) as the precursor of SRO Fe (hydr)oxides (Fig. 3A, C and D) [29,43]. By comparison, drainage negatively affected both soluble phenols and 0.5Fe_o_ + Al_o_ (mainly via decreasing Fe(II)) in the *Sphagnum* wetlands, thereby showing an overall negative effect on bound OC% (Fig. 3B–D). Overall, the SEMs explained 70% and 44% of the drainage-induced variations in bound OC% in the non-*Sphagnum* and *Sphagnum* wetlands, respectively. The results further supported our hypothesis that drainage-induced decline of *Sphagnum* initiated cascading effects on soil properties including SRO Fe and Al (hydr)oxides, leading to decreasing bound OC% in the *Sphagnum* wetlands, in contrast to drainage-enhanced metallic stabilization of soil carbon in the non-*Sphagnum* wetlands.

### Distinguishing the effects of drainage and land-use change on wetland SOC

The above results demonstrate that long-term drainage increased surface SOC content and bound OC% in most (two-thirds) of our examined non-*Sphagnum* wetlands, standing in contrast to drainage-induced decreases of SOC contents based on a recent global meta-analysis [12]. This discrepancy may be related to different land-use regimes after drainage. The examined wetlands in our study were mostly located in Natural Reserves and had rarely been disturbed after ditch construction. By comparison, a large proportion of previous studies on wetland drainage were based on reclaimed areas, where agriculture or pasture management rather than drainage may destabilize soil carbon [53–55]. To reconcile the discrepancy potentially related to different land-use regimes, we compared the effects of ‘drainage only’ and ‘reclamation after drainage’ on SOC contents and bound OC% at our sites and in the published studies.

**Figure 4.**
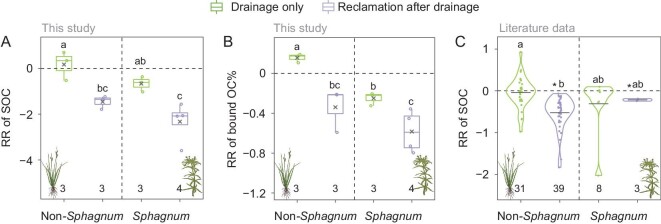
Distinguishing the effects of drainage and land-use change on SOC. Response ratio (RR) of SOC and bound OC% in soils experienced ‘drainage only’ or ‘reclamation after drainage’ relative to the paired waterlogged soils, based on (A and B) our paired soil survey and (C) the literature data. Abbreviations are defined in Fig. 2. Solid line and cross in the box mark the median and mean of each data set, respectively. The upper and lower ends of boxes denote the 0.25 and 0.75 percentiles, respectively. The upper and lower whisker caps denote the maximum and minimum values, respectively. Shapes of the violin represent distribution of data. The dots and solid lines in the violin plot denote the raw data and mean of each data set, respectively. Lowercase letters indicate different levels among groups (*P* < 0.05; one-way ANOVA). Asterisk denotes a significant difference with zero (*P* < 0.05; t-test). The numbers below the box or violin indicate the number of observations.

We first surveyed reclaimed areas adjacent to (within 200–1000 m of) our examined wetlands, and identified seven sites that had been used for agriculture (mainly for maize and soybean) after drainage (three near non-*Sphagnum* and four near *Sphagnum* wetlands; also see Data S1). We calculated the RR of SOC content and bound OC% for the surface soils (0–20 cm) under ‘reclamation after drainage’ relative to the paired waterlogged soils, and compared with the RR of the corresponding drained areas (‘drainage only’). As aforementioned, ‘drainage only’ induced an overall positive response of bound OC% and SOC content (except one site) in the non-*Sphagnum* wetlands (RR > 0). However, ‘reclamation after drainage’ decreased both SOC and bound OC% (Fig. 4A and B), suggesting that reclamation-induced negative effect on SOC and bound OC% surpassed the increase caused by drainage. In contrast, ‘drainage only’ induced an overall negative response (RR < 0) of both SOC content and bound OC% in the *Sphagnum* wetlands, and the negative effect was even more pronounced by ‘reclamation after drainage’ (*P* < 0.05; Fig. 4A and B). Thus, the decrease caused by reclamation after drainage was substantial and cannot be ignored. The loss of bound OC was mainly attributed to a sharp decrease (by 46% relative to the waterlogged soils) of 0.5Fe_o_ + Al_o_ after reclamation at all sites (Fig. S4). This was likely associated with the increase in oxide crystallinity after prolonged exposure to oxygen and/or the loss of reactive metals upon tillage and irrigation, etc. [43,53], although tillage is beneficial for soil aggregate formation in most agricultural soils.

To complement the comparison based on our paired sites, we compiled literature data on SOC content changes under ‘drainage only’ and ‘reclamation after drainage’, yielding 81 pairs of published measurements in the surface soils (0–30 cm) from 70 non-*Sphagnum* and 11 *Sphagnum* wetlands (Fig. S8 and Data S2). Bound OC was not included, given the paucity of reports. Based on the literature data, ‘drainage only’ did not significantly affect SOC contents in either type of wetland (the average RR was not significantly different from zero; T test; *P* > 0.05; Fig. 4C), whereas ‘reclamation after drainage’ significantly decreased SOC content in both types of wetlands (*P* < 0.05; Fig. 4C). Hence, both our paired survey and the literature data revealed that reclamation rather than drainage had a strong negative effect on the surface SOC content of drained wetlands, suggesting that the effect of drainage on wetland soil carbon should be treated with caution depending on the subsequent land-use type.

### Drainage-induced changes of SOC and bound OC stocks along depths

**Figure 5.**
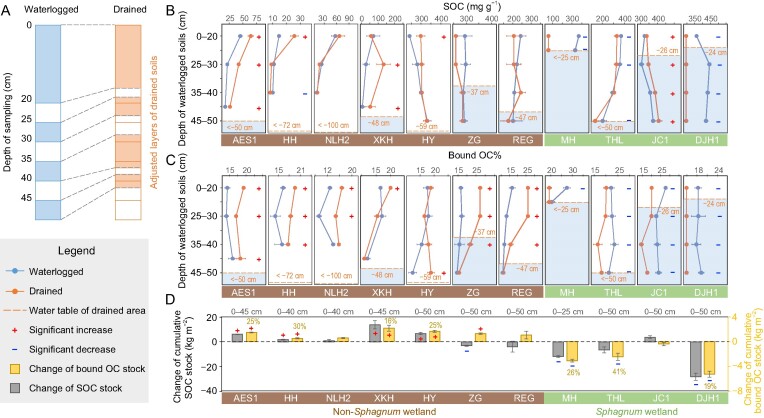
Changes in SOC and bound OC after wetland drainage across depths. (A) Schematic representation of the equivalent ash mass method (example of THL site). (B and C) Changes of SOC and bound OC% after wetland drainage based on adjusted depths according to the equivalent ash mass method. (D) Changes of SOC and bound OC stocks after wetland drainage in each profile. Abbreviations are defined in Fig. 2. For THL in (A), ground subsidence and soil compaction led to bulk density increases and thus the sampling depth did not correspond to equivalent soil layers. The dashed line and number in (B) and (C) indicates the water-table level of drained soils. Percentage in (D) indicates the ratio of bound OC stock change to SOC stock change, which is calculated only if SOC and bound OC stocks are changing in the same direction and the change is significant. Error bars indicate standard error of mean (*n* = 3). The plus and minus signs represent significant increase and decrease after drainage compared to the waterlogged soils, respectively (*P* < 0.05; two-way ANOVA).

Given that drainage may affect soils beyond the surface layer, we further examined drainage-induced changes in SOC and bound OC content and stocks along 66 soil profiles (mostly 0–50 cm) in 11 pairs of waterlogged vs. drained soils (*n* = 3) at our surveyed sites (including seven non-*Sphagnum* and four *Sphagnum* wetlands; Table S1). Because the conventional quantification of carbon pools at fixed depths (i.e. based on sampling depths) was subject to error due to substantial changes of bulk density caused by ground subsidence and soil compaction after drainage [56–58], we applied the equivalent ash mass method [59] (see details in Methods) for carbon pool comparisons. This method was based on the assumption that the mineral component (mostly silicate minerals) of soil (i.e. ash mass) remained unaffected by changes in bulk density over time, and can thus provide reliable estimates of OC changes in organics-rich soils in response to environmental changes [58,59]. Although drainage-induced oxidation may increase metal (mainly Fe and Al) oxides in the soil, we calculated that metal oxides constituted ∼5% (Data S3) of ash mass in our soils based on the measured contents of dithionite-extractable Fe and Al (Fe_d_ and Al_d_), and their effect on ash mass variations was negligible. Hence, we first adjusted and matched the layers of the drained soils to attain the ‘equivalent ash mass’ against the sampling layers of the waterlogged soils (Fig. 5A, Fig. S5 and Data S4). Subsequently, we analyzed SOC, bound OC and 0.5Fe_o_ + Al_o_ contents in three to four layers (in most cases 0–20, 25–30, 35–40 and 45–50 cm, depending on sample availability) of the waterlogged soils and in the corresponding adjusted layers of the drained soils (Data S5). We then calculated and compared the content and stocks of both bound OC and SOC in the drained vs. waterlogged soils at the fixed ash mass accordingly (Data S6).

In most of our examined non-*Sphagnum* wetlands (except ZG), the water-table was close to or lower than –50 cm (Fig. 5B) in the drained area at the time of sampling (which was the rainy season with a relatively high water-table). The SOC content increased or remained unchanged in the drained relative to the waterlogged soils in the same adjusted layer (Fig. 5B). However, bound OC% increased significantly in most layers above the water-table (except HY; *P* < 0.05; Fig. 5C). Differences in bound OC% between the drained and waterlogged soils seemed to wane or disappear toward or below the water-table of the drained area (except HY), implying that drainage effect on bound OC% was dependent on the water-table depth. At HY (in Zoige), drainage-induced increase of bound OC% was only significant in the deep layer (45–50 cm), possibly because large fluctuations of water-table in the drained wetlands of Zoige (between –20 cm to –80 cm in May to August according to continuous *in-situ* monitoring) [60] prevented a clear manifestation of the drainage effect at the surface.

In contrast to the non-*Sphagnum* wetlands, SOC content decreased in almost all layers along three out of four examined *Sphagnum* wetland profiles after drainage (except JC1; *P* < 0.05; Fig. 5B), even though some of the layers were well below the water-table of the drained area at the time of sampling (at THL and DJH1). Bound OC% also decreased in almost all of the examined layers of the drained relative to waterlogged *Sphagnum* wetlands except a submerged layer (20–25 cm) of MH (*P* < 0.05; Fig. 5C). This result suggested that drainage-induced *Sphagnum* replacement had far-reaching consequences on SOC and bound OC loss beyond the depth of water-table decline. Notably, SOC contents increased throughout the profile of JC1 despite bound OC% decreases after drainage, due to increased POC accumulation (*P* < 0.05; Fig. S6A). This site had the highest clay content and belowground biomass after drainage among our investigated *Sphagnum* wetlands (*P* < 0.05; Fig. S6B and C). Shifts from rootless *Sphagnum* to deep-rooted *Carex* (Table S1) may have greatly enhanced root litter inputs and POC accrual in this clay-rich soil that facilitated POC protection via aggregate formation [42].

To demonstrate bound OC variations in relation to SOC stock changes, we estimated stocks of bound OC and SOC for all examined soil profiles based on the ‘equivalent ash mass’ layers. In four of the seven non-*Sphagnum* wetlands (AES1, HH, XKH and HY) showing significant increases in both bound OC and SOC stocks (Fig. 5D), bound OC stock increased by an average of 1.5 ± 0.2 kg m^−2^, contributing to 24.0 ± 1.6% of the corresponding SOC stock increases (by 7.0 ± 1.5 kg m^−2^; Fig. 5D). The bound OC contribution to SOC stock increases was higher than bound OC% (15.7 ± 0.3%; *P* < 0.05; one-way ANOVA) in the corresponding waterlogged soils, suggesting that the bound OC increase was larger than that of POC in the same soil and promoted SOC accrual at these sites. Neither SOC nor bound OC stocks changed at NLH2. In the other two non-*Sphagnum* wetlands (ZG and REG), bound OC stock also increased (by 6–58%; Fig. 5D) with considerable increases of SRO Fe and Al (hydr)oxides in the soil (by 88%–102%; Fig. S7A), while SOC stock decreased (by 8%–33%). This result indicated opposite responses of bound OC vs. POC to drainage, where enhanced metallic protection of OC partly compensated for POC loss in these wetlands. Conversely, drainage decreased bound OC stock by 3.6 ± 0.5 kg m^−2^ in three out of four surveyed *Sphagnum* wetlands (MH, THL and DJH1; Fig. 5D), which contributed to 19%–41% of the SOC stock decreases (by 15.6 ± 3.5 kg m^−2^) therein. At JC1, bound OC stock did not change, while SOC stock increased (although not significantly) by 3.7 ± 1.1 kg m^−2^, likely due to the aforementioned reasons (high root input and POC accumulation; Fig. S6). These results indicated that although bound OC stock did not dictate the direction of SOC variations following wetland drainage, it showed consistently contrasting changes in the non-*Sphagnum* vs. *Sphagnum* wetlands, sometimes in opposite directions to the unbound OC (e.g. POC) pools. Hence, drainage-induced change of bound OC was an important mechanism regulating SOC stock variations in wetlands.

### Implications for wetland SOC stabilization and management

This study presents the first regional-scale assessment of the drainage effect on metal-OC interactions in wetlands, an under-investigated mechanism mediating wetland carbon response to water-table drawdown. Employing a nationwide, pairwise survey of drained wetlands across China (Fig. 1 and Table S1), we demonstrate divergent changes in the metallic protection of SOC (i.e. bound OC%) in non-*Sphagnum* vs. *Sphagnum* wetlands following long-term drainage, delineate pathways driving the contrasting bound OC% variations and reveal its relation to SOC stock changes. There are some differences in the region we studied here compared with other regions. Our studied wetlands were noted to have a relatively thin layer of peat, which differs from many boreal ombrotrophic bogs (e.g. in North America and Europe) [61]. Metal-OC interactions in peat with low mineral content warrant further study to investigate the applicability of the ‘iron gate’ mechanism therein. Our sites were also mostly dominated by *Sphagnum* and herbaceous plants both before and after drainage, whereas drainage-induced expansion of woody plants has been reported in many northern wetlands [39]. As phenolic compounds have a strong impact on the speciation and interaction of Fe [44], how metal-OC interactions vary under woody plants rich in phenolic compounds also awaits investigation. Clearly, drainage-induced carbon stabilization by reactive metals warrants further evaluation across a wider range of wetlands globally. Our study highlights an underappreciated mechanism (metallic protection of SOC) in wetlands that creates resilience of carbon stores to drought. Those results have two important implications for understanding and predicting wetland carbon dynamics under a shifting hydrological regime.

First, drainage can increase bound OC% in a range of non-*Sphagnum* wetlands with distinct climatic, geological and edaphic characteristics (Fig. 2B and D), primarily due to oxidation-induced increases of reactive Fe and Al (hydr)oxides in the soil (Fig. 2E and Fig. 3A–D) [19,20,23]. Soil acidification (likely due to Fe oxidation) [45,47,49] and increasing soluble phenols [50,51] after drainage also played a (relatively minor) part (Fig. 2E, and Fig. 3A and D). These results fall in line with the ‘iron gate’ mechanism [23] and suggest that drainage-enhanced metallic stabilization of soil carbon is prevalent in non-*Sphagnum* wetlands. However, this emergent mechanism may be obscured by land-use effect on soil carbon following wetland reclamation after drainage [12,62]. Both our paired survey and a synthesis of the literature data indicate that reclamation rather than drainage has a strong negative effect on SOC and bound OC of drained wetlands (Fig. 4). Hence, metallic stabilization of soil carbon needs to be quantified in the context of well-defined land-use regimes after wetland drainage [9]. It is also notable that although bound OC stock increases did not dictate the direction of SOC variations following wetland drainage (e.g. at ZG; Fig. 5B–D), they compensated for the loss of unbound components (e.g. POC). Given the relatively slower turnover of mineral-associated OC than POC [21,22], bound OC variations may play a more important role in SOC dynamics over longer timescales. Alternatively, redox oscillations in wetlands can also potentially lead to bound OC release via Fe reduction [52,63,64]. The stability of bound OC thus awaits evaluation under water-table fluctuations. It is nevertheless clear that drainage-associated metallic protection represents an important but previously overlooked mechanism regulating SOC dynamics in wetlands and one that deserves better recognition in wetland carbon modeling and prediction. The increase of bound OC may result from mineral preservation of increased fresh plant inputs [23] and/or from the transformation of native SOC, e.g. from unbound to bound OC resulting from enhanced physiochemical protection by minerals and/or from increased microbial processes [26]. The source of the increased bound OC deserves further investigation by employing more specific analytical measurements, such as biomarkers.

Second, in contrast to non-*Sphagnum* wetlands, long-term drainage consistently weakened metal-OC interactions in *Sphagnum* wetlands following the replacement of *Sphagnum* by herbaceous plants (Fig. 2B and D, and Table S1) [19,36]. The contrasting response of bound OC to drainage in these two types of wetlands was mainly attributed to the unique function of *Sphagnum* as an efficient ‘rust engineer’ which maintains a high level of bound OC in waterlogged wetlands [19] by enhancing the formation of SRO Fe and Al (hydr)oxides owing to its acidic, phenolic metabolites [29,36]. Decreasing bound OC contributed to a substantial loss of SOC in most of the drained *Sphagnum* wetlands (Fig. 5C and D), underscoring the high vulnerability of carbon pools in *Sphagnum* wetlands to drainage. The divergent sensitivity of carbon in *Sphagnum-* (or moss) vs. vascular-plant-dominated wetlands has recently been underscored in a meta-analysis of greenhouse gas emissions under warming-induced drought [30]. Our study suggests that the contrasting response of metal-OC interaction to drainage (drought) may be a key mechanism underpinning the divergent sensitivity of OC pools in these two types of wetlands. Given the remarkable carbon stocks in global *Sphagnum* wetlands [33–35], it is imperative to prioritize the protection of existing *Sphagnum* wetlands to avoid substantial carbon loss. In addition, as carbon degradation in drained *Sphagnum* wetlands is closely associated with vegetation shifts (Fig. 3B and Table S1), cultivation and restoration of the functional plant (*Sphagnum*) may be a vital part of wetland restoration other than waterlogging [19].

In all, metallic stabilization of wetland SOC is clearly a widespread but often overlooked mechanism and one that is heavily influenced by vegetational shifts. Incorporating this new knowledge with a readily measurable parameter related to the transformation of reactive Fe and Al (hydr)oxides into wetland soil carbon models would allow us to greatly improve the prediction of wetland SOC dynamics under shifting hydrological regimes.

## METHODS

Detailed descriptions of methods are available in the Supplementary Data.

## Supplementary Material

nwae178_Supplemental_Files

## Data Availability

All data (including compiled data) supporting the findings are available online in the Supplementary Data.
