# Supplementary material for: Metallic protection of soil carbon: divergent drainage effects in Sphagnum vs. non-Sphagnum wetlands
Source: Natl Sci Rev. 2024 May 20;11(11):nwae178. doi: 10.1093/nsr/nwae178 (PMC11493091; doi:10.1093/nsr/nwae178)
Supplement: nwae178_Supplemental_Files [file nwae178_supplemental_files.zip › Supplementary Data-new.pdf]

Supplementary Data for

Metallic protection of soil carbon: Divergent drainage effects in *Sphagnum* vs. non-*Sphagnum* wetlands

Chengzhu Liu[1,2,3,†], Yunpeng Zhao[1,2,3,†], Lixiao Ma[1,2,3], Guoqing Zhai[1,2,3], Xingqi Li[1,2,3], Chris Freeman[4], Xiaojuan Feng[1,2,3,*]

[1]State Key Laboratory of Vegetation and Environmental Change, Institute of Botany, Chinese Academy of Sciences, Beijing 100093, China.

[2]China National Botanical Garden, Beijing 100093, China.

[3]University of Chinese Academy of Sciences, Beijing 100049, China.

[4]School of Natural Sciences, Bangor University, Bangor LL57 2UW, UK.

†These authors contributed equally to this work.

*Corresponding author: Xiaojuan Feng (xfeng@ibcas.ac.cn); Tel: +86 10 6283 6162.

This file includes:

Methods

Figs. S1 to S8

Tables S1 to S2

Legends for Supplementary Data S1 to S6

Other Supplementary Materials for this manuscript include the following:

Supplementary Data S1 to S6

METHODS

Survey of typical drained wetlands in China

We surveyed 32 drained (by ditching for 15–55 years) wetlands relative to their waterlogged pristine counterparts across China in the summer (July-August) of 2019-2022, including 18 non-*Sphagnum* and 14 *Sphagnum* wetlands (Fig. 1 and Table S1). The surveyed wetlands represented typical wetlands in China developed on the Great and Small Khingan Mountains, Sanjiang Plain, Changbai Mountain, subalpine basins in Central China, Qinghai-Tibet Plateau, Yunnan-Guizhou Plateau, Yangtze and Yellow River basins. Twenty-four out of the 32 sites were located in Natural Reserves, where the drained areas were not reclaimed after ditch construction. The other eight sites were not reclaimed, either. These sites span significant gradients in climatic, geological and edaphic characteristics. The mean annual temperature (MAT) ranges from –5.5 to 18.9 °C, and mean annual precipitation (MAP) ranges from 150 to 1,635 mm [1]. At each sampling site, the drained area (~20 m from the drainage ditch) was paired with the pristine waterlogged area nearby (100–800 m from the drained area). The water-table declined to 20–100 cm below the soil surface during the sampling period (typically the monsoon season; Table S1) in the drained areas, but remained above or near soil surface in the waterlogged areas. Drainage shifted the dominant species from *Carex* and *Polygonum,* etc. in the waterlogged area to *Carex, Deyeuxia,* and *Kobresia,* etc. in the non-*Sphagnum* wetlands, and from *Sphagnum* to *Carex* and *Deyeuxia,* etc. in the *Sphagnum* wetlands (Table S1).

In each wetland, four replicated quadrats of 1×1 m were established at ~50 m intervals along paralleled transects perpendicular to the drainage ditch in the waterlogged and drained areas, respectively (Fig. 1A). The dominant species were identified within each quadrat, and plant aboveground biomass were collected by cutting within an embedded 50 cm × 50 cm box. The topsoil underneath was sampled (0–20 cm; for a total of 252 samples) with PVC pipes (diameter of 7 or 10 cm, depth of 25 cm), after the aboveground litter (if any) was removed. A small aliquot of the topsoil was immediately mixed in 0.5-M hydrochloric acid (HCl) in the field to extract acid-extractable Fe(II) (described below).

To compare the effect of 'drainage only' and 'reclamation after drainage', we further surveyed reclaimed areas adjacent to our sampled wetlands (distance between 200 to 1000 m), and identified seven areas that had been used for agriculture after wetland drainage (three near non-*Sphagnum* and four near *Sphagnum* wetlands; Fig. 1A and Table S1). In the past 20–40 years,

these areas had been drained for agriculture (mainly for maize and soybean, except one site for rice) [1] with the aboveground biomass harvested every year. In the July-August of 2022–2023, we collected surface soils (0–20 cm; n = 3) from the reclaimed areas using the same protocol as described above.

In addition to surface soils, 11 pairs (n = 3 each) of our surveyed wetlands (including seven non-*Sphagnum* and four *Sphagnum* wetlands) were selected to investigate drainage effects along depths (Fig. 1A and Table S1). After removing the aboveground biomass and litter (if any), soil cores were sampled in duplicates using a specialized corer (diameter of 5 cm) designed to minimize soil compaction. The soil was sampled to a depth of 25 cm at MH (where soil was frozen beneath), and to 50 cm at the other 10 sites. Both sets of soil cores were sliced at 5-cm intervals for the measurement of bulk density, belowground biomass and other soil properties, respectively. Water-table depth was measured manually by placing a PVC pipe into a dipwell (created using a soil corer with a diameter of 2.5 cm or the hole left after soil core sampling), followed by a tape measure. Measurements were conducted at each plot of each site, and the average water-table depth was used in this study. All of the soil and plant samples were transported (cooled by ice bags) to the laboratory immediately. The plant samples were dried to constant weight at 65°C and weighed for aboveground biomass. Belowground biomass for each layer was obtained by washing soils over a 1-mm sieve and was then freeze-dried and weighed. The soil samples were freeze-dried and sieved (< 1 mm) with roots removed by hand before further analysis. Bound OC, SRO Fe and Al (hydr)oxides and basic soil properties for the waterlogged soils (topsoils) have been reported in [1] (Data S1). All parameters for the drained soils and soil profiles were analyzed in this study.

Bulk soil properties

Soil bulk density was determined by drying fresh soils collected by the specialized corers at 105˚C for about 24 h to a constant weight. To minimize damage to the HY wetland (located in a long-term monitoring site), bulk density of HY soils was measured for the surrounding soils (< 1 m from the sampling area) and the mean values were used here (n = 6 for waterlogged soils; n = 3 for drained soils). Soil pH was measured at a soil:water ratio of 1:5 (w:v). SOC contents were determined on an elemental analyzer (Vario EL III; Elementar, Hanau, Germany) after removal of inorganic carbon by acidification [2]. Soluble phenols were extracted by mixing 1 g of freeze-dried soil with 20 mL of Milli-Q water for 2 h. After centrifugation, the supernatant was filtered through

0.45-μm filters and quantified using the Folin-Ciocaltu method [3] at 750 nm by a Multi-Mode Microplate Reader (synergy Mx, BioTek Instruments Inc., USA). Soil ash content was determined on 1 g of freeze-dried, ground soils (< 1 mm) based on mass loss after heating in a muffle furnace at 500 °C for 5 h [4]. To measure soil texture, sieved (< 2 mm) and freeze-dried soils (0.15–0.5 g) were first treated with hydrogen peroxide at 120°C for approximately 9 h to remove OC until no bubbles were produced. Then, the samples were heated at 275°C for 1 h with 10 ml of 3-M HCl to remove inorganic carbon, with pH adjusted to 6~9 and dispersed in 10 ml of sodium hexametaphosphate at 300°C for 30 min. Particle size distribution was determined using the laser diffraction method [5] on a Laser Particle Size Analyzer (Mastersizer 2000). Clay was defined as particles smaller than 2 μm. Note that Fe(II) and soluble phenols were unfortunately not analyzed in AES1 and NLH2.

To examine the unbound OC (i.e., POC) in relation to SOC increases at JC1, we separated particulate organic matter (POM) according to [1]. Briefly, dried and sieved soils (< 1 mm) were wet-sieved through a 53-μm sieve. The size fraction of < 53 μm was sonicated with sodium polytungstate solution (density of 1.6 g cm^{-3}) for 1 min to separate light fraction and mineral-associated organic matter (MAOM). Light fraction and MAOM were repeatedly rinsed with Milli-Q water until the electric conductivity was <50 μ S cm^{-3}. Both POM (including size fractions of > 53 μm and the light fraction) and MOAM were collected, freeze-dried, weighed and ground. The OC content of POM was analyzed as described previously.

Metal species and bound OC

To probe Fe transformation during wetland drainage, acid-extractable Fe(II) was extracted immediately by 0.5-M HCl at a soil:solution ratio of 1:5 (w:v) in the field to minimize oxidation and measured by the ferrozine absorbance method [6]. After centrifugation, the supernatant was mixed with 5-mM ferrozine solution, and Fe(II) was determined by absorbance at 562 nm on an ultraviolet and visible spectrometer (Shimadzu UVÀ 2550). A standardized calibration curve for ferrous ammonium sulfate (0–50 mg L^{-1}) was created using the same method outlined above. Oxalate-extractable Fe and Al (Fe_o and Al_o), representing poorly crystalline or SRO Fe and Al (hydr)oxides that play an important role in SOC stabilization [7,8], were extracted by ammonium oxalate-oxalic acid from freeze-dried soils for 4 h in the dark [9]. Dithionite-extractable Fe and Al (Fe_d and Al_d), including SRO and crystalline Fe and Al not bound in silicates, was extracted using

the CBD method [10]. Dissolved metals in the filtered suspensions (< 0.45 μm) were quantified on an inductively coupled plasma-optical emission spectrometer (ICP-OES; iCAP 6300, Thermo Scientific, USA). The calibration solutions were prepared by diluting of certified standard solutions of Fe and Al (1000 μg mL^{-1}). The blank control was evaluated by deionized water. To ensure quality control procedures, the measurements were performed in triplicate. To evaluate the total effect of reactive Fe_o and Al_o, we calculated the weight-normalized contents of Fe_o and Al_o as "$0.5Fe_o+Al_o$" to normalize the atomic mass difference between Fe_o and Al_o for graphing and statistical purposes [11].

Bound OC was examined using a modified CBD method [10]. Briefly, two aliquots of freeze-dried soils (0.25 g) were extracted with 15 mL of CBD solution (containing 0.27 M trisodium citrate, 0.11 M bicarbonate and 0.25 g of dithionite) at 80°C in a water bath for 15 min twice. After centrifugation, the CBD-treated soil residues were rinsed with sodium chloride (NaCl; 1 M) solution four times. Another aliquot of dry soils (0.25 g) was also extracted with NaCl (0.25 M) in tandem as a control with another buffer solution (containing 1.6 M NaCl and 0.11 M sodium bicarbonate). All soil residues after extraction were dried, fumigated with HCl, and subjected to OC measurement on an elemental analyzer. Bound OC% was determined as:

$$\text{Bound OC\%} = (OC_{NaCl} - OC_d) / SOC \times 100\% \quad (1)$$

where OC_{NaCl} and OC_d are the OC content of NaCl- and CBD-treated soil residues, respectively; SOC refers to the OC content of bulk soil.

Soil OC and bound OC stocks

The conventional soil carbon stock quantification based on fixed depth intervals is subject to errors due to ground subsidence and soil compaction after drainage [12,13]. Hence, we applied the equivalent ash mass method to evaluate SOC and bound OC stock changes [4] in 11 selected wetlands. This method is similar to the equivalent soil and mineral mass methods used in previous studies, which can provide reliable estimates of the carbon changes in organic soils in response to permafrost thaw and land management [4,13,14]. Given that ash is a relatively stable component of soil over time, this method assumes that there is no gain or loss of ash or exogenous ash deposition in the soil surface along wetland drainage [4]. However, it should be recognized that drainage-induced metal oxidation (mostly Fe and Al) may cause an increase of ash mass, which seems to disobey the assumption of equivalent ash mass method, and would lead to an

underestimate or overestimate on soil carbon pool. To rule out this possibility, we estimated an upper limit of Fe and Al oxides in our soils based on contents of Fe_d and Al_d (representing both SRO and crystalline phases) [10], assuming that Fe and Al oxides mainly consisted of goethite and gibbsite (with the lowest fraction of Fe (62.9%) and Al (36.0%) among the commonly observed Fe and Al oxides in soils). Then we calculated the ratio of Fe and Al oxides to ash mass. The result showed that the total contents of Fe and Al oxides were similar ($P > 0.05$; one-way ANOVA; Fig. S7B and Data S3) between the waterlogged (21.5 ± 0.9 mg g^{-1} soil; n = 69) and drained soils (21.3 ± 0.8 mg g^{-1} soil; n = 96) and contributed to 5.0 ± 0.3% (n = 165; Fig. S7C) of ash mass in our soils. Hence the effect of Fe and Al oxide content on ash mass can be negligible, and our estimation is reliable based on equivalent ash mass method.

Based on the assumption of equivalent ash mass method [4], we first calculated the ash mass (AM; kg m^{-2}) and soil mass (SM; kg m^{-2}) of each core segment of the waterlogged and drained soils, respectively:

$$AM_i = (A_i \times BD_i \times L_i) / 100 \quad (2)$$

$$SM_i = (BD_i \times L_i) / 100 \quad (3)$$

where A_i is the ash concentration (g kg^{-1}), BD_i is the bulk density (g cm^{-3}), and L_i is the length (cm) of the core segment i (raw data in Data S4). The mean of AM_i of the waterlogged soils at each site is specified as the reference ash mass (i.e., equivalent ash mass, EAM) for the corresponding drained soils. Similarly, we calculated SOC or bound OC stock (XP; kg m^{-2}) of each core segment for the waterlogged and drained soils, respectively:

$$XP_i = (X_i \times BD_i \times L_i) / 100 \quad (4)$$

where X_i is the SOC or bound OC content (g kg^{-1}) of the core segment i. We further adjusted the depth as well as stocks of SOC and bound OC for the drained soils to attain the EAM of the corresponding waterlogged soils (XP_{EAM}; kg m^{-2}) by linear interpolation within the range of data points for the deepest core segment (i) (Fig. S5):

$$XP_{EAMi} = (XP_{i-1}) \frac{AM_{i-1} - EAM_{i-1}}{AM_{i-1} - AM_{i-2}} + (XP_i) \frac{EAM_i - AM_{i-1}}{AM_i - AM_{i-1}} \quad (5)$$

where AM_{i-2}, AM_{i-1}, AM_i are the ash mass (kg m^{-2}) of the core segment (i–2), (i–1) and i, respectively. XP_{i-1}, XP_i is the bound OC pool (kg m^{-2}) of the core segment (i–1) and i, respectively. Similarly, we adjusted soil mass (SM; kg m^{-2}) for the drained soils to attain the EAM of the corresponding waterlogged soils (SM_{EAM}; kg m^{-2}) by linear interpolation using equation (5). The adjusted SOC content was calculated by SOC stock divided by the soil mass within each adjusted

EAM segment. The adjusted bound OC% was calculated by bound OC stock divided by the SOC stock within each adjusted EAM segment. Then, we used the SOC or bound OC% at each sampled depth of the waterlogged soils to compare with that at the adjusted layer of the corresponding drained soils (Fig. 5A; adjusted data in Data S6). The calculations are illustrated in Fig. S5 with specific data from the THL site.

Taking THL site as an example (Fig. 5A), three subsoil layers (25–30, 35–40, 45–50 cm) in the waterlogged soils were analyzed for SOC and bound OC along with the topsoil (0–20 cm). For the drained soils, six layers (0–20, 20–25, 25–30, 30–35, 35–40 and 40–45 cm) were chosen for SOC or bound OC measurement according to the results of ash measurement, i.e., the depth to attain the EAM of the corresponding waterlogged soils. Note that bulk density, ash and SOC were analyzed in each layer of surface soils (i.e., 0–5, 5–10, 10–15, 15–20 cm; Data S5), while bound OC% and Fe_o, Al_o for these layers were from topsoil (0–20 cm; mean of 4 replicated plots; Data S1). When assessing stocks of SOC and bound OC of the whole profiles, we used measurements from adjacent depths to estimate stocks for the depths not measured, for example, using data of 25–30 cm to estimate stocks of 20–30 cm.

Meta analysis of literature data

To supplement the comparison based on our paired sites, we further conducted a synthesis of literature data on SOC changes under ‘drainage only’ and ‘reclamation after drainage’. Bound OC was not included due to the paucity of reports. We searched all peer-reviewed articles using Google Scholar and the Web of Science with the following keywords: “wetland” OR “peatland” OR “bog” OR “fen” OR “marsh” OR “swamp” AND “drainage” OR “water table decline” OR “drained” OR “managed” OR “land use” AND “SOC” OR “soil organic carbon”. Data were selected according to the following three criteria: (1) only references that contained SOC in both drained (‘drainage only’ or ‘reclamation after drainage’) and waterlogged counterparts (areas) were included; (2) the locations, wetland types and land use histories were described; (3) the means, standard errors (deviation) and sample sizes in the treatment and control groups can be calculated or extracted from the original publications. Wetlands were classified into *Sphagnum* and non-*Sphagnum* according to the dominant species in the waterlogged areas. In total, our database comprised of 81 pairs of measurements in the surface soils (0–30 cm) from 70 non-*Sphagnum* wetlands and 11 *Sphagnum* wetlands (Fig. S8 and Data S2). Note that 13 pairs of total soil carbon (possibly

including inorganic carbon) were included to increase the number of data. Considering that these soils had pH < 6 (except one study with a pH of 7.9), we reason that the contribution or influence from inorganic carbon was minor.

Statistical analysis

Data analysis was performed using the R software (version 4.3.1; R Core Team, 2023). The fixed effects of treatment (waterlogged vs. drained), species (non-*Sphagnum* vs. *Sphagnum* wetlands), and their interactions on SOC and bound OC% were analyzed by linear mixed effects models, with site served as random factor, using the *lme* function in R “nlme” package [15]. Subsequently, we performed multiple comparisons with Tukey’s test, using the *glht* function in R “multcomp” package [16], to test differences of SOC and bound OC% among treatment (waterlogged vs. drained) and species (non-*Sphagnum* vs. *Sphagnum* wetlands) ($P < 0.05$). Differences between treatments (waterlogged vs. drained) at each site were analyzed by one-way ANOVA. For profile samples, two-way ANOVA was used to examine the effects of drainage and depth for each site, followed by one-way ANOVA in the presence of interactive effects. Relationships between the tested variables were explored using Spearman correlation (for non-normally distributed data). Differences were considered to be significant at a level of $P < 0.05$. Partial correlation analysis was conducted to exclude the direct effect of aboveground biomass on bound OC% with $0.5Fe_o+Al_o$ being controlled for, and *vice versa*.

To compare soil’s response to drainage for different wetlands, i.e., in the drained relative to the paired waterlogged soils, RR was calculated for individual site [17]:

$$\mathrm{RR} = \ln\left(\frac{\mathrm{X_t}}{\mathrm{X_c}}\right) = \ln(\mathrm{X_t}) - \ln(\mathrm{X_c}) \quad (6)$$

where $\mathrm{X_t}$ and $\mathrm{X_c}$ are the means for each soil variable in the drained and paired waterlogged soils at each site, respectively. The variance (v) of the logarithmic effect size was calculated as follows:

$$v = \left(\frac{S_c^2}{N_c X_c^2}\right) + \left(\frac{S_t^2}{N_t X_t^2}\right) \quad (7)$$

where $\mathrm{S_c}$ and $\mathrm{S_t}$ are the standard deviations of $\mathrm{X_t}$ and $\mathrm{X_c}$, and $\mathrm{N_c}$ and $\mathrm{N_t}$ are the sample size of data. The weighting function was calculated based on the reciprocal of the variance in individual RRs:

$$w_{ij} = \frac{1}{v} \quad (8)$$

We further consolidated the RR of individual site to evaluate site-weighted response (RR_{++}) for the non-*Sphagnum* and *Sphagnum* wetlands, respectively. The weighted response ratio (RR_{++})

was calculated from the individual RR_{ij} (i = 1, 2,…, m; j = 1, 2,…, k) by pairwise comparison between the drained and waterlogged groups, where m is the number of groups and k is the number of comparisons in the ith group:

$$RR_{++} = \frac{\Sigma_{i=1}^{m}\Sigma_{j=1}^{k_i} w_{ij} RR_{ij}}{\Sigma_{i=1}^{m}\Sigma_{j=1}^{ki} w_{ij}} \quad (9)$$

The standard error (S) of RR_{++} was estimated as follows:

$$S(RR_{++}) = \sqrt{\frac{1}{\Sigma_{i=1}^{m}\Sigma_{j=1}^{ki} w_{ij}}} \quad (10)$$

The 95% confidence interval (95% CI) was $RR_{++} \pm 1.96\ S(RR_{++})$. If the 95% CI did not overlap with zero, the drainage response was considered to be significant.

To identify the main predictors for bound OC%, we conducted a random forest model using "randomForest" package in R [18]. The accuracy importance measure was computed for each tree and averaged over the forest (1000 trees). Four predictors were included in the model, including $0.5Fe_o+Al_o$, Fe(II), soluble phenols, and pH, ranked in the order of importance based on mean increase in mean square error (%IncMSE). The higher the %IncMSE, the more important the predictors were in affecting bound OC%.

To explore the direct and indirect pathways driving bound OC% changes after wetland drainage, we conducted pathway analysis by SEM using the dataset of 252 surface soils. We first considered an *a priori* model that included all reasonable pathways (Fig. S3). In the *a priori* model, drainage induced the variation in other factors. Soluble phenols, $0.5Fe_o+Al_o$ and soil pH showed direct effects on bound OC%, while Fe(II) had an indirect effect. Then, we sequentially eliminated non-significant pathways or added pathways based on the residual correlations. The procedure was repeated until the model showed sufficient fitting with $P > 0.05$. The goodness of fit of the SEM was evaluated using Fisher's C statistic, Akaike information criterion (AIC), and the whole-model P value [19]. The SEM-related analysis was performed using the "piecewiseSEM" package [19].

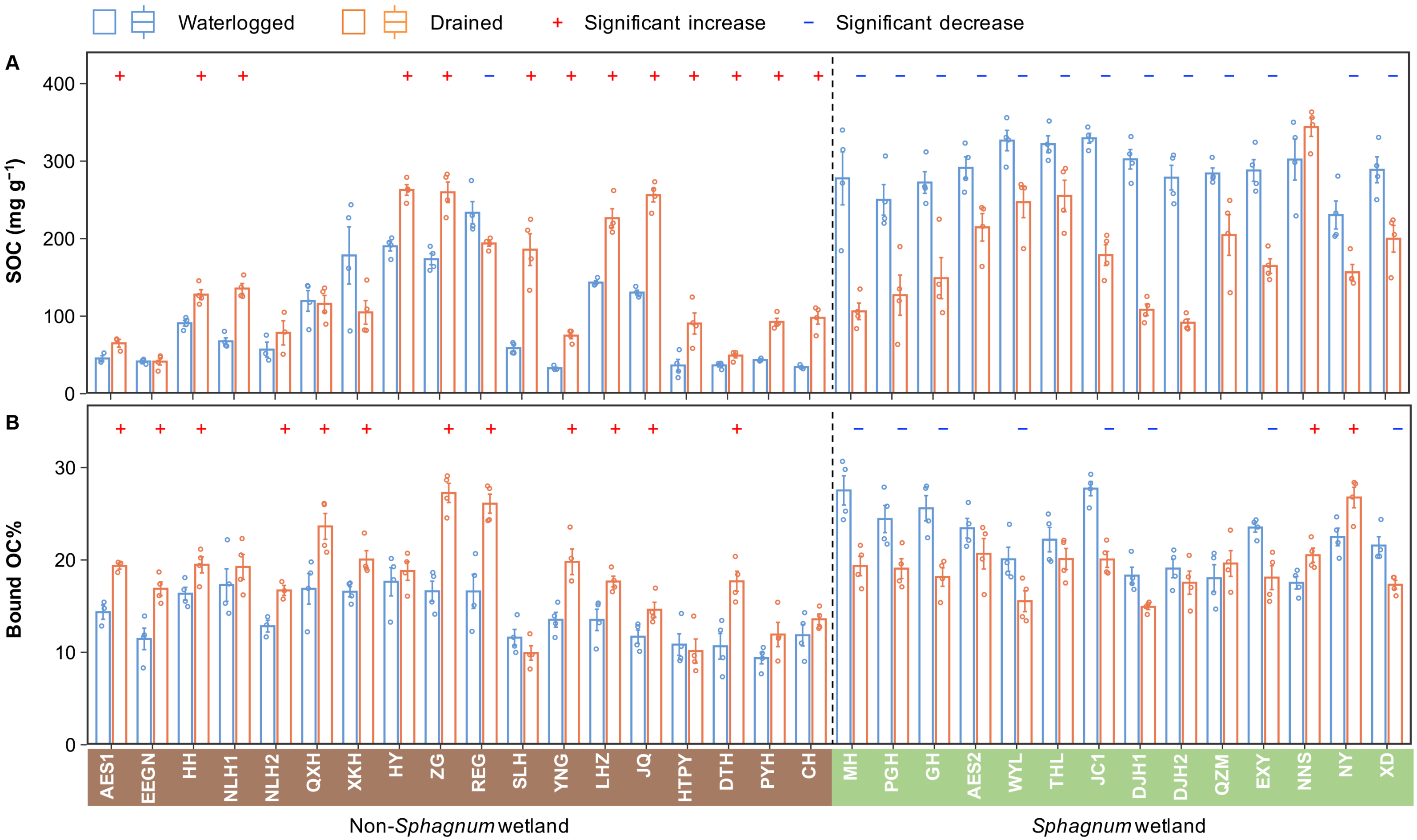

Fig. S1. Contents of SOC (**A**) and bound OC% (**B**) in the drained and waterlogged areas of each wetland. Abbreviations are defined in Fig. 2. The signs of plus and minus represent significant increase and decrease, respectively ($P < 0.05$; one-way ANOVA). Error bars represent standard error of mean (n = 3 for AES1 and NLH2; n = 4 for the remaining sites).

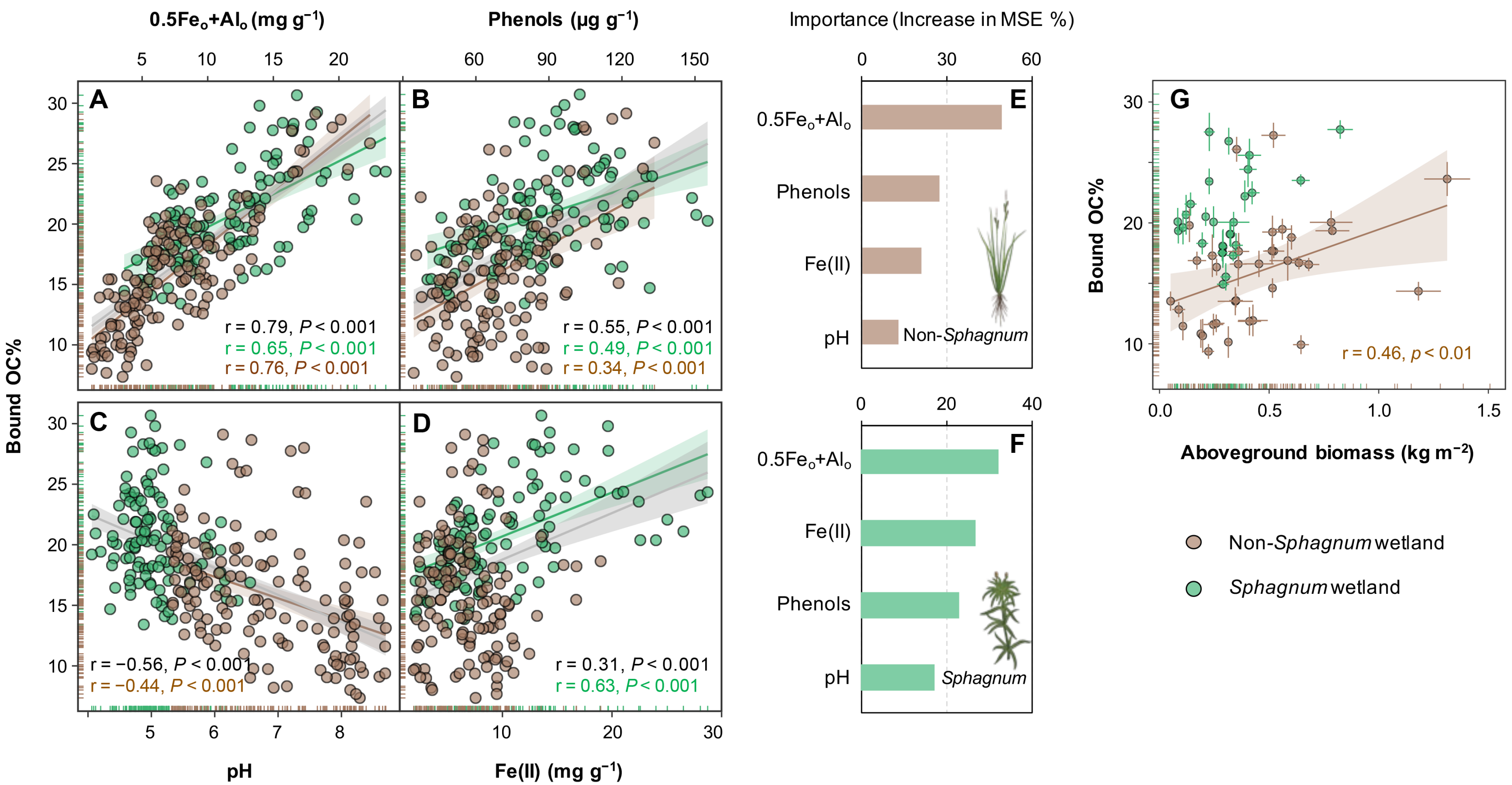

0.5Fe$_o$+Al$_o$ (mg g^{-1})
Phenols (μg g^{-1})
Importance (Increase in MSE %)
A
B
C
D
E
F
G
Bound OC%
r = 0.79, P < 0.001
r = 0.65, P < 0.001
r = 0.76, P < 0.001
r = 0.55, P < 0.001
r = 0.49, P < 0.001
r = 0.34, P < 0.001
r = −0.56, P < 0.001
r = −0.44, P < 0.001
r = 0.31, P < 0.001
r = 0.63, P < 0.001
pH
Fe(II) (mg g^{-1})
0.5Fe$_o$+Al$_o$
Phenols
Fe(II)
pH
Non-Sphagnum
Sphagnum
r = 0.46, p < 0.01
Aboveground biomass (kg m^{-2})
Non-Sphagnum wetland
Sphagnum wetland

Fig. S2. Relationships of bound OC% with potential drivers in surface soils. Spearman's correlations of bound OC% with $0.5Fe_o+Al_o$ (**A**), soluble phenols (**B**), soil pH (**C**), Fe(II) (**D**), and aboveground biomass (**G**). Relative importance of potential variables in influencing bound OC% revealed by random forest model for non-*Sphagnum* (**E**) and *Sphagnum* wetlands (**F**), respectively. Abbreviations are defined in Fig. 2. %IncMSE: percentage of increase of mean square error of variables, is used to estimate the importance of these predictors, and higher %IncMSE values imply more important predictors. Brown, green, and black lines represent significant correlations for non-*Sphagnum, Sphagnum* wetlands and all data, respectively ($P < 0.05$). The shaded areas represent the 95% confidence intervals. Note that aboveground biomass is only measured in three plots, while bound OC% is measured in four plots.

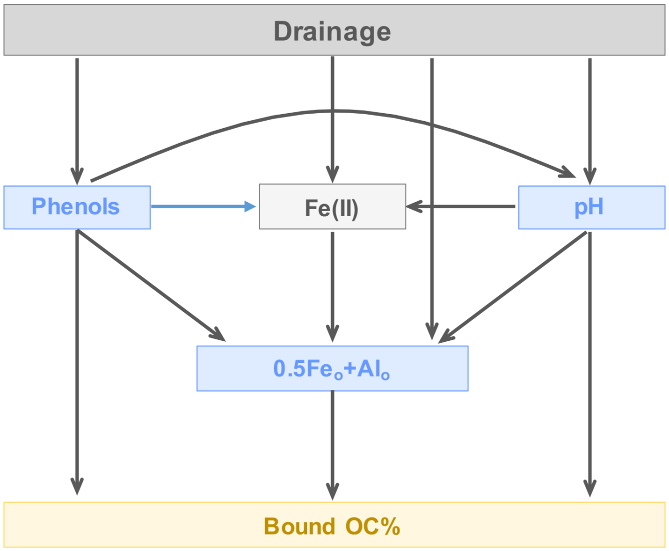

Fig. S3. A *priori* model for the structural equation model (SEM) showing the direct and indirect effects of wetland drainage on bound OC%. Abbreviations are defined in Figure 2. Arrows indicate flows of causality based on knowledge.

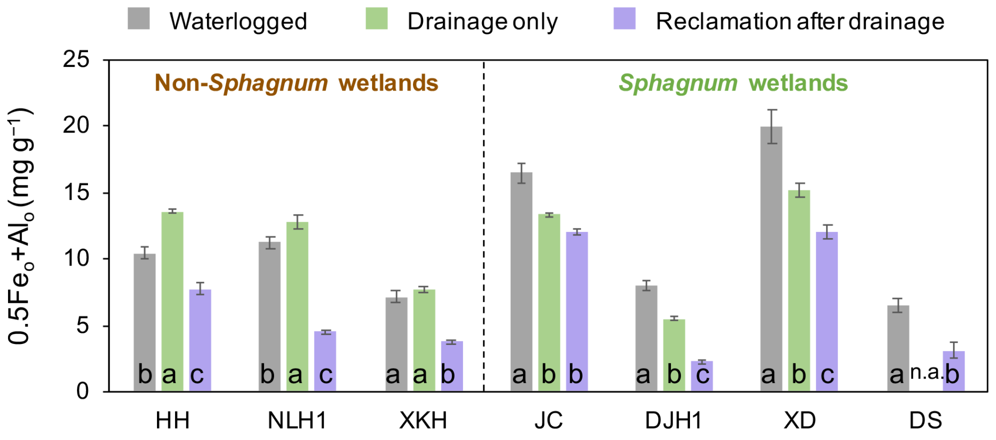

Fig. S4. Comparisons of 0.5Fe_o+Al_o between three land-use regimes in surface soils. Error bars represent standard error of mean (n = 3 for reclamation; n = 4 for waterlogged and drainage only). Abbreviations are defined in Fig. 2. Lowercase letters indicate different levels among groups (p < 0.05; one-way ANOVA). Note that data in DS is from [1].

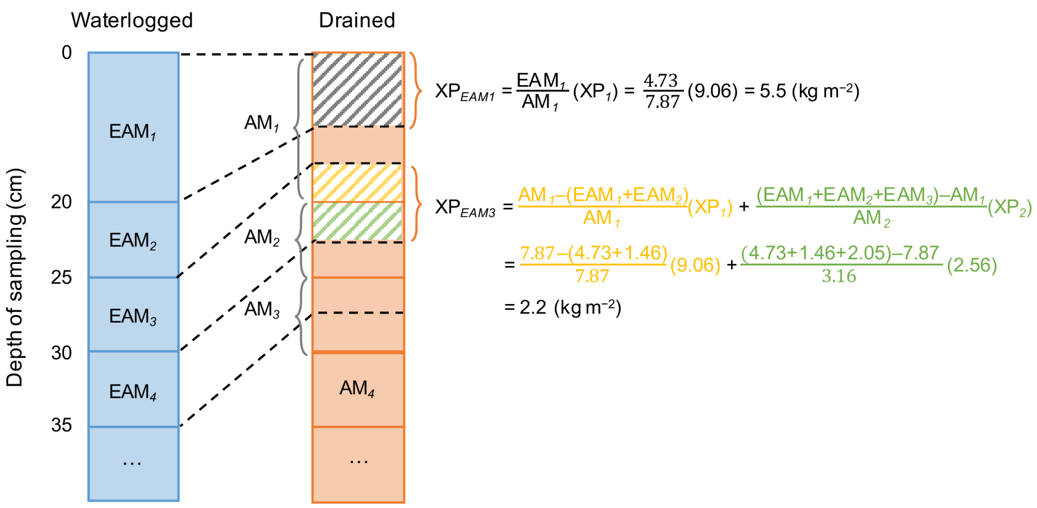

Core segment	Depth of sampling (cm)	Mean of EAM_i (kg m^{-2})	Mean of AM_i (kg m^{-2})	XP_i (kg m^{-2})
1	0–20	4.73	7.87	9.06
2	20–25	1.46	3.16	2.56
3	25–30	2.05	3.91	3.14

Fig. S5. Calculation process of soil carbon pool of drained soils with specific data from the THL site based on the equivalent ash mass method. AM_i, the ash mass of each core segment for drained soils; EAM_i, AM of waterlogged soils in each core segment, which is specified as the reference ash masses for the corresponding drained soils. XP_i, the SOC stock of each core segment for drained soils; XP_{EAMi}, the adjusted SOC stock of each core segment for drained soils to attain the EAM of corresponding waterlogged soils. Similarly, we adjusted soil mass (SM; kg m^{-2}) for the drained soils to attain the EAM of the corresponding waterlogged soils (SM_{EAM}; kg m^{-2}). The adjusted SOC content was calculated by SOC stock divided by the soil mass within each adjusted EAM segment. The adjusted bound OC% was calculated by bound OC stock divided by the SOC stock within each adjusted EAM segment. Then, we used the SOC or bound OC% at each sampled depth of the waterlogged soils to compare with that at the adjusted layer of the corresponding drained soils. Pools of POC, $0.5Fe_o+Al_o$ as well as belowground biomass for drained soils were adjusted using the same methods.

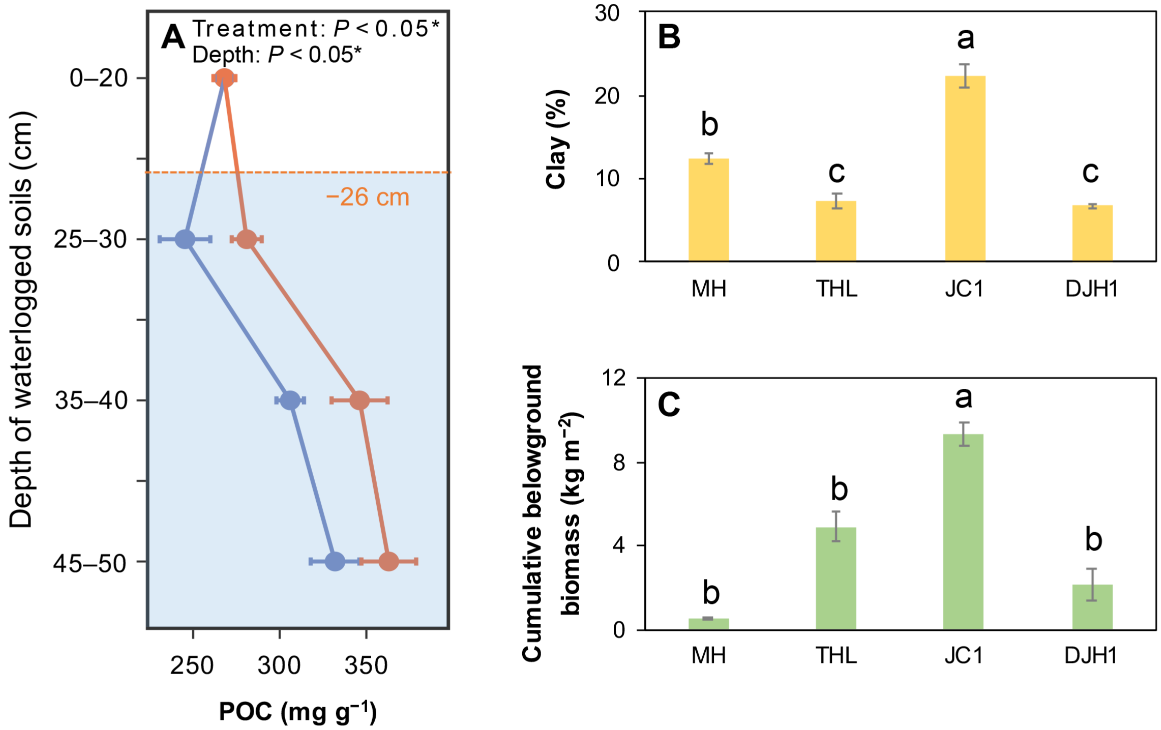

Fig. S6. Plant and soil properties at JC1. (**A**) Changes of POC content after wetland drainage across depths based on equivalent ash mass method; (**B**) clay content in surface soils (0–20 cm) of waterlogged area; (**C**) cumulative belowground biomass in the top 50 cm of the drained area based on equivalent ash mass method. Two-way ANOVA was used to examine the effects of drainage and depth in (**A**). The orange dash line and number in (**A**) indicate the water-table level of the drained soils. Lowercase letters on top of the bars in (**B**) and (**C**) indicate different levels among sites ($P < 0.05$; one-way ANOVA).

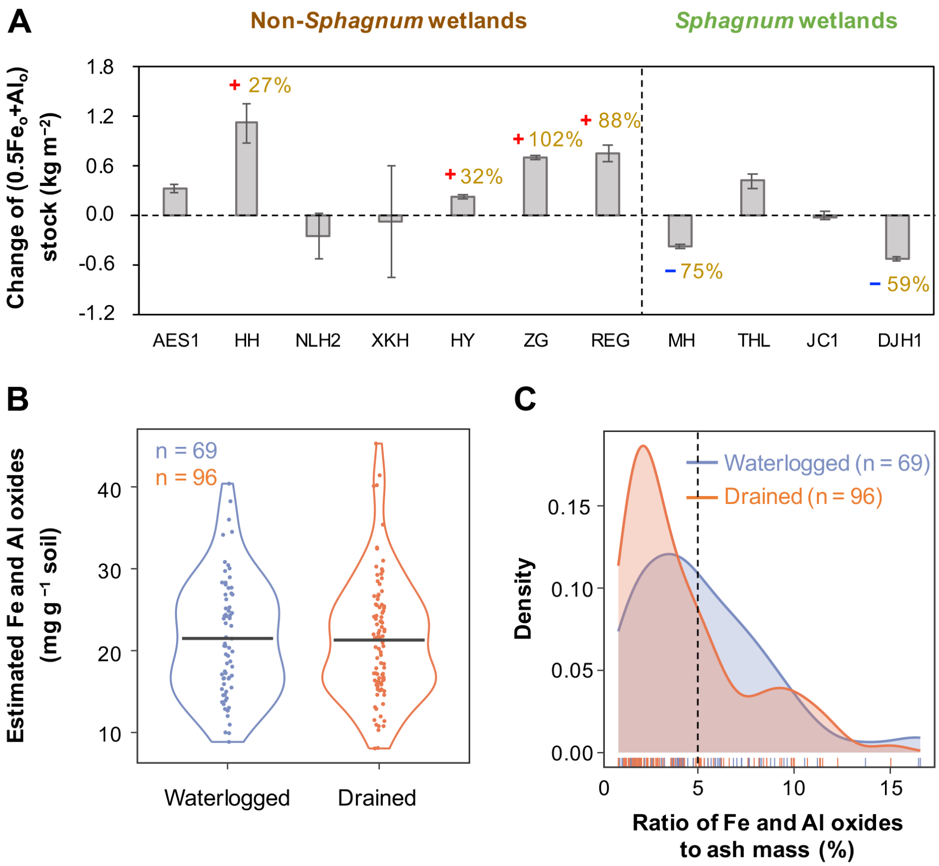

Fig. S7. Change of (0.5Fe$_o$+Al$_o$) stock after wetland drainage in the whole profile based on equivalent ash mass method (**A**); Fe and Al oxides content (**B**) and the distribution of ratios of Fe and Al oxides to ash mass (**C**). Abbreviations are defined in Fig. 2. Error bars in (**A**) represent standard error of mean (n = 3). The signs of plus and minus represent significant increase and decrease after drainage compared to waterlogged soils, respectively ($P < 0.05$; one-way ANOVA). Percentage indicates relative increase or decrease of (0.5Fe$_o$+Al$_o$) stock after drainage, which is calculated only if (0.5Fe$_o$+Al$_o$) stock changes significantly. Shapes of the violin represent distribution of data. Solid line in the box marks the mean of each dataset. Dots denote raw data. The dash line in (**C**) denotes the mean of all data.

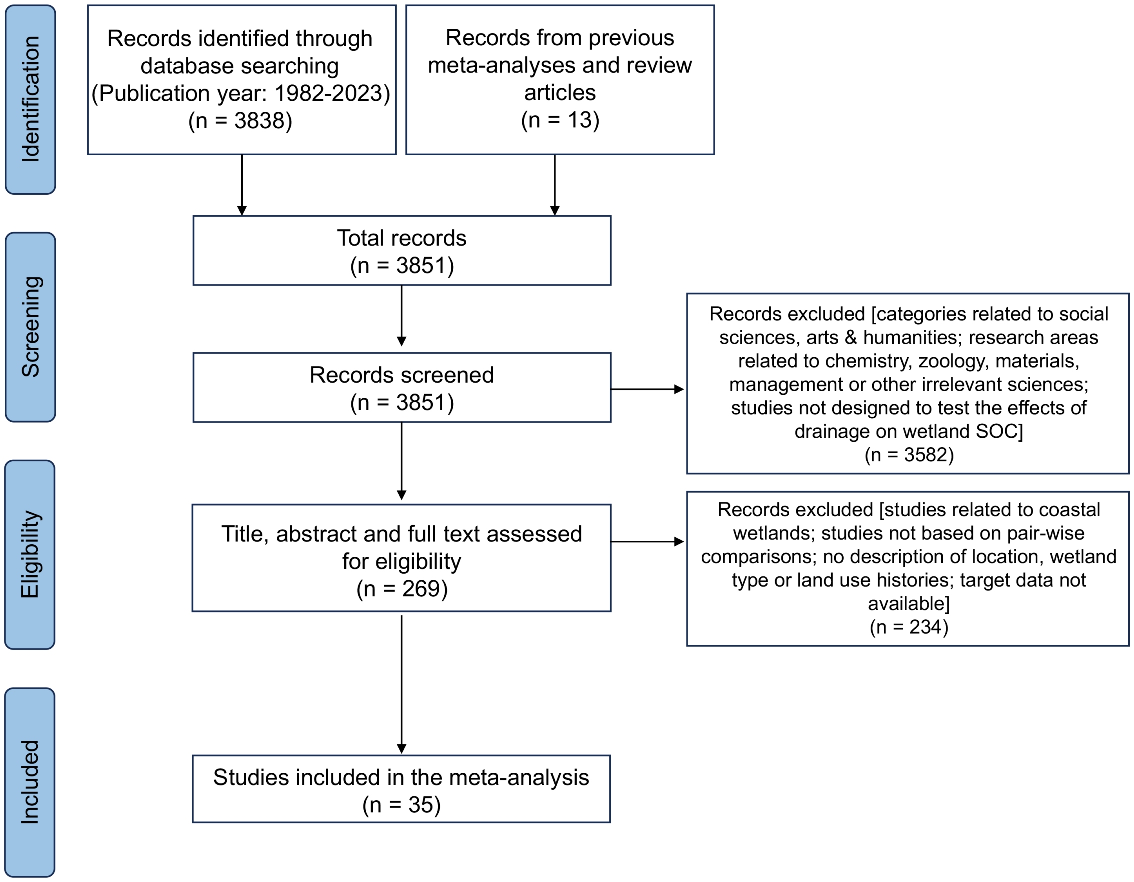

Fig. S8. Article selection process using Preferred Reporting Items for Systematic Reviews (PRISMA) guidelines.

Table S1. Overview of the sampling locations and main variables across 32 pairs of waterlogged and drained wetlands in China.

Site	Type of waterlogged wetlands	Latitude (°C)	Longitude (°C)	MAT (°C)	MAP (mm)	Drainage Time (year)	Water-table (cm)	Dominated plant		Core depth (cm)
								Waterlogged	Drained	
AES1	non-*Sphagnum*	47.4	120.2	–3.1	451	30	–50	*Carex*	*Carex*	0–45
HH[b]	non-*Sphagnum*	47.8	133.6	1.9	585	30	–72	*Carex*	*Deyeuxia*	0–40
NLH1[b]	non-*Sphagnum*	47.2	133.5	2.3	546	25	–83	*Carex*	*Deyeuxia*	n.a.
NLH2	non-*Sphagnum*	47.2	133.4	2.3	546	45	< –100	*Carex*	*Deyeuxia*	0–40
QXH	non-*Sphagnum*	46.7	132.0	2.4	551	30	< –100	*Carex*	*Deyeuxia*	n.a.
XKH[b]	non-*Sphagnum*	45.4	132.3	3	750	35	< –48	*Carex*	*Deyeuxia*	0–45
HY	non-*Sphagnum*	33.1	102.6	2.9	860	45	–59	*Carex*	*Carex*	0–50
ZG	non-*Sphagnum*	33.5	102.9	1	690	40	–37	*Carex*	*Kobresia*	0–50
REG	non-*Sphagnum*	33.7	103.0	1	690	30	–47	*Carex*	*Carex*	0–50
SLH	non-*Sphagnum*	37.3	100.1	–0.3	380	45	–40	*Blysmus*	*Leymus*	n.a.
YNG	non-*Sphagnum*	38.6	99.3	1	420	35	< –90	*Carex*	*Festuca*	n.a.
LHZ	non-*Sphagnum*	37.6	101.3	–1.1	490	45	–35	*Carex*	*Kobresia*	n.a.
JQ	non-*Sphagnum*	38.8	94.4	3.9	150	25	–30	*Carex*	*Leymus*	n.a.
EEGN	non-*Sphagnum*	50.3	120.2	–2.5	240	35	–30	*Carex*	*Calamagrostis*	n.a.
HTPY	non-*Sphagnum*	38.5	106.2	8.5	200	15	–20	*Typha*	*Acorus*	n.a.
DTH	non-*Sphagnum*	29.1	112.9	16.5	1250	55	–40	*Polygonum*	*Carex*	n.a.
PYH	non-*Sphagnum*	29.1	116.7	17	1500	25	–30	*Phragmites*	*Carex*	n.a.
CH	non-*Sphagnum*	26.9	104.3	10.5	950	20	–30	*Phragmites*	*Carex*	n.a.

Table S1. continued

Site	Type of waterlogged wetlands	Latitude (°C)	Longitude (°C)	MAT (°C)	MAP (mm)	Drainage Time (year)	Water-table (cm)	Dominated plant		Core depth (cm)
								Waterlogged	Drained	
MH	*Sphagnum*	52.9	122.7	–5.5	460	45	< –25 [a]	*Sphagnum*	*Juncus*	0–25
PGH	*Sphagnum*	52.8	124.6	–2.4	463	30	–25	*Sphagnum*	*Carex*	n.a.
GH	*Sphagnum*	51.2	121.7	–5	424	40	–15	*Sphagnum*	*Deyeuxia*	n.a.
AES2	*Sphagnum*	47.4	120.6	–3.1	451	25	–50	*Sphagnum*	*Carex*	n.a.
WYL	*Sphagnum*	48.6	129.4	–1.1	585	55	–25	*Sphagnum*	*Carex*	n.a.
THL	*Sphagnum*	48.4	129.2	–1	500	25	< –50 [a]	*Sphagnum*	*Deyeuxia*	0–50
JC[b]	*Sphagnum*	42.3	126.4	3.3	1054	35	–26	*Sphagnum*	*Carex*	n.a.
JC1	*Sphagnum*	42.3	126.4	3.3	1054	35	–26	*Sphagnum*	*Carex*	0–50
DJH1[b]	*Sphagnum*	31.5	110.0	7.2	1560	30	–24	*Sphagnum*	*Carex*	0–50
DJH2	*Sphagnum*	31.5	110.0	7.2	1560	30	–33	*Sphagnum*	*Juncus*	n.a.
QZM	*Sphagnum*	30.0	109.8	13.7	1635	50	< –100	*Sphagnum*	*Carex*	n.a.
EXY	*Sphagnum*	29.7	108.8	14	1555	40	–30	*Sphagnum*	*Juncus*	n.a.
NNS	*Sphagnum*	26.1	104.8	13.5	1350	25	–35	*Sphagnum*	*Polytrichum*	n.a.
NY	*Sphagnum*	26.7	105.5	13.6	1243	35	< –100	*Sphagnum*	*Juncus*	n.a.
XD[b]	*Sphagnum*	25.6	103.0	14.9	921	15	–20	*Sphagnum*	*Dactylis*	n.a.

Abbreviations: n.a., not available; MAT, mean annual temperature; MAP, mean annual precipitation.

[a]MH and THL are located in permafrost regions, and the depths of the frozen soil layer correspond to their water tables.

[b]Agricultural soils adjacent to these wetlands are collected to investigate the effect of reclamation.

Table S2. Values of r for the partial correlation analysis between aboveground biomass, SRO Fe and Al (hydr)oxides and bound OC% ($p < 0.05$). Aboveground biomass is only measured in three plots, while SRO Fe and Al (hydr)oxides and bound OC is measured in four plots. Hence, mean values of plot replicates are used.

Tested variable	Controlled variable	
	$0.5Fe_o+Al_o$	Aboveground biomass
$0.5Fe_o+Al_o$	na	0.81
Aboveground biomass	ns	na

$0.5Fe_o+Al_o$, weight-normalized contents of oxalate-extractable iron (Fe_o) and aluminum (Al_o); na, not applicable; ns, not significant.

Supplementary Data S1. (separate file). Raw data of plant and soil properties for our surveyed wetlands.

Supplementary Data S2 (separate file). Information on sampling sites and raw data of literature data.

Supplementary Data S3 (separate file). Estimated contents of Fe and Al oxidates.

Supplementary Data S4 (separate file). Raw data for the equivalent ash mass method.

Supplementary Data S5 (separate file). Raw data of soil profiles.

Supplementary Data S6 (separate file). Adjusted data of drained soils based on the equivalent ash mass method.

REFERENCES

1. Zhao Y *et al.* *Sphagnum* increases soil's sequestration capacity of mineral-associated organic carbon via activating metal oxides. *Nat Commun* 2023; **14**: 5052.
2. Harris D, Horwath WR and van Kessel CD. Acid fumigation of soils to remove carbonates prior to total organic carbon or carbon-13 isotopic analysis. *Soil Sci Soc Am J* 2001; **65**: 1853-6.
3. Box JD. Investigation of the Folin-Ciocalteau phenol reagent for the determination of polyphenolic substances in natural waters. *Water Res* 1983; **17**: 511-25.
4. Plaza C *et al.* Direct observation of permafrost degradation and rapid soil carbon loss in tundra. *Nat Geosci* 2019; **12**: 627-31.
5. Ryzak M and Bieganowski A. Methodological aspects of determining soil particle-size distribution using the laser diffraction method. *J Plant Nutr Soil Sci* 2011; **174**: 624-33.
6. Stookey LL. Ferrozine-a new spectrophotometric reagent for iron. *Anal Chem* 1970; **42**: 779-81.
7. Anthony TL and Silver WL. Mineralogical associations with soil carbon in managed wetland soils. *Glob Change Biol* 2020; **26**: 6555-67.
8. Hall SJ and Thompson A. What do relationships between extractable metals and soil organic carbon concentrations mean? *Soil Sci Soc Am J* 2022; **86**: 195-208.
9. Bhattacharyya A *et al.* Iron speciation in peats: chemical and spectroscopic evidence for the co-occurrence of ferric and ferrous iron in organic complexes and mineral precipitates. *Org Geochem* 2018; **115**: 124-37.
10. Lalonde K *et al.* Preservation of organic matter in sediments promoted by iron. *Nature* 2012; **483**: 198-200.
11. Wagai R, Kajiura M and Asano MR. Iron and aluminum association with microbially processed organic matter via meso-density aggregate formation across soils: organo-metallic glue hypothesis. *Soil* 2020; **6**: 597-627.
12. Leifeld J, Müller M and Fuhrer JJ. Peatland subsidence and carbon loss from drained temperate fens. *Soil Use Manag* 2011; **27**: 170-6.
13. von Haden AC, Yang WH and DeLucia E. Soils' dirty little secret: depth-based comparisons can be inadequate for quantifying changes in soil organic carbon and other mineral soil properties. *Glob Change Biol* 2020; **26**: 3759-70.

14. Lee J *et al*. Determining soil carbon stock changes: simple bulk density corrections fail. *Agric., Ecosyst Environ* 2009; **134**: 251-6.

15. Pinheiro J *et al*. R Core Team, 2020. Nlme: Linear and Nonlinear Mixed Effects Models. R Package Version 3.1-148. URL: https://CRAN. R-project.org/package=nlme.

16. Hothorn T, Bretz F and Westfall P. Simultaneous inference in general parametric models. *Biom J* 2008; **50**: 346-63.

17. Hedges LV, Gurevitch J and Curtis PS. The meta-analysis of response ratios in experimental ecology. *Ecology* 1999; **80**: 1150-6.

18. Breiman L. Random forests. *Machine Learning* 2001; **45**: 5-32.

19. Lefcheck JS. piecewiseSEM: Piecewise structural equation modelling in r for ecology, evolution, and systematics. *Methods Ecol Evol* 2016; **7**: 573-9.
